# Tunable Alginate-Polyvinyl Alcohol Bioinks for 3D Printing in Cartilage Tissue Engineering

**DOI:** 10.3390/gels10120829

**Published:** 2024-12-14

**Authors:** Alexandra Hunter Aitchison, Nicholas B. Allen, Kishen Mitra, Bijan Abar, Conor N. O’Neill, Kian Bagheri, Albert T. Anastasio, Samuel B. Adams

**Affiliations:** 1 Department of Orthopaedic Surgery, Duke University Health System, Durham, NC 27710, USA; alexandra.aitchison@duke.edu (A.H.A.); nicholas.allen@duke.edu (N.B.A.); bijan.abar@duke.edu (B.A.); conor.n.oneill@duke.edu (C.N.O.); albert.anastasio@duke.edu (A.T.A.); 2 Department of Mechanical Engineering, Duke University, Durham, NC 27710, USA; kishen.mitra@duke.edu

**Keywords:** tissue engineering, bioink, alginate, polyvinyl alcohol, PVA, hydrogel, 3D bioprinting, 3D scaffold, cartilage, matrix

## Abstract

This study investigates 3D extrusion bioinks for cartilage tissue engineering by characterizing the physical properties of 3D-printed scaffolds containing varying alginate and polyvinyl alcohol (PVA) concentrations. We systematically investigated the effects of increasing PVA and alginate concentrations on swelling, degradation, and the elastic modulus of printed hydrogels. Swelling decreased significantly with increased PVA concentrations, while degradation rates rose with higher PVA concentrations, underscoring the role of PVA in modulating hydrogel matrix stability. The highest elastic modulus value was achieved with a composite of 5% PVA and 20% alginate, reaching 0.22 MPa, which approaches that of native cartilage. These findings demonstrate that adjusting PVA and alginate concentrations enables the development of bioinks with tailored physical and mechanical properties, supporting their potential use in cartilage tissue engineering and other biomedical applications.

## 1. Introduction

Cartilage tissue engineering has advanced significantly with 3D bioprinting technologies, enabling the precise creation of scaffolds for tissue regeneration. Among various biomaterials, polyvinyl alcohol (PVA) and alginate have emerged as promising candidates for bioink formulations. PVA is valued for its mechanical strength and cytocompatibility [1,2,3,4], while alginate is valued for its biocompatibility and rapid ionic gelation [5,6,7,8,9]. The combination of these two materials in bioinks offers a unique opportunity to create scaffolds with enhanced properties suitable for cartilage tissue engineering [9,10].

The structure of the alginate–PVA hydrogel is primarily formed through a combination of physical and chemical interactions. Alginate undergoes ionic cross-linking to form a 3D network that provides structural integrity to the gel [11]. PVA enhances the network through hydrogen bonding and the formation of crystalline regions within its matrix [12,13]. The chemical interactions between alginate and PVA are primarily mediated by hydrogen bonds formed between the hydroxyl groups of PVA and the carboxylate groups of alginate [14]. The synergy between ionic and hydrogen bonding contributes to the dual-network structure of the gel where the alginate offers structural integrity and the PVA contributes to the elasticity and mechanical strength of the hydrogel [15].

Recent studies have demonstrated that alginate-based bioinks with tunable degradation rates can effectively support the chondrogenic differentiation of mesenchymal stem/stromal cells (MSCs), leading to the formation of hyaline-like tissue rich in type II collagen [8,16,17]. However, alginate-based gels lack inherent mechanical strength. Double-crosslinked alginate hydrogels have exhibited superior mechanical properties and stability, although their mechanical strength still falls short of native cartilage [9]. To address this limitation, researchers have explored combining alginate with PVA to enhance both the physical and biological properties of hydrogels. For instance, Bichara et al. demonstrated that porous PVA–alginate constructs improved mechanical properties and supported neocartilage formation [3]. Similarly, Wei et al. examined a limited range of alginate (7–14%) and PVA (0–7%) formulations, providing valuable insights into their mechanical properties and printability under controlled conditions [18]. These studies highlight the promise of combining PVA and alginate, as the addition of PVA has been shown to further enhance both physical and biological properties of alginate hydrogels, underscoring the combined potential of PVA and alginate in tissue-engineering applications [3,10,19].

Despite progress in understanding PVA–alginate composites, most studies characterize single formulations [20], or alter only one component [1], leaving the effects of varying both PVA and alginate concentrations on key properties—such as mechanical strength, degradation, and swelling—largely unexplored. Addressing this gap is essential to fully harness the potential of alginate–PVA hydrogels for cartilage tissue engineering. To date, no study has comprehensively examined how a wide range of PVA and alginate concentrations interact to influence these properties. This information will provide a foundation for guiding the formulation of hydrogels tailored to the specific physical and mechanical requirements of 3D printing applications in cartilage tissue engineering and beyond.

We hypothesize that combining PVA and alginate in bioink formulations enables systematic modulation of key hydrogel properties—swelling behavior, degradation rates, and the elastic modulus—through precise concentration adjustments within the printable range. Furthermore, we propose that a composition involving intermediate levels of PVA and higher alginate concentrations may result in scaffolds with enhanced mechanical stability, controlled degradation, and reduced swelling. These properties are expected to better mimic the mechanical and functional characteristics of native cartilage, facilitating their application in 3D bioprinting for cartilage tissue engineering and repair. To test this hypothesis, we systematically investigate how varying concentrations of alginate and PVA influence these key properties. This study aims to elucidate these relationships to guide the development of bioink formulations tailored for cartilage tissue engineering and further advance the translational potential of alginate–PVA bioinks for cartilage repair.

## 2. Results and Discussion

### 2.1. Overview

**Figure 1:**
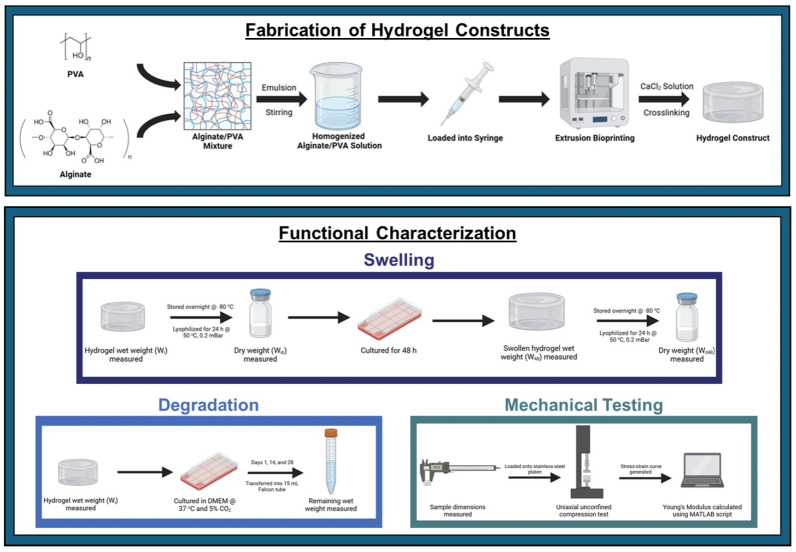
Schematic overview of hydrogel fabrication and functional characterization. The top panel illustrates the process of hydrogel synthesis, combining bioprinting technology and crosslinking to produce stable constructs. The bottom panel summarizes the key methods used to evaluate hydrogel performance, including swelling behavior, mechanical properties, and degradation over time.

This study investigates the fabrication and characterization of 3D-printed hydrogels, focusing on the effects of alginate and PVA concentrations on key properties including swelling behavior, degradation, and mechanical performance (Figure 1).

### 2.2. Swelling

**Figure 2:**
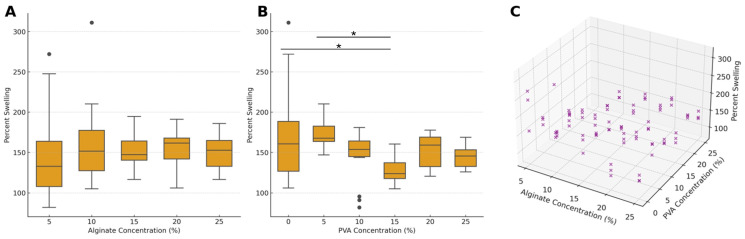
Percent swelling behavior of hydrogel samples in response to varying concentrations of alginate and PVA. (**A**) Box-and-whisker plots showing percent swelling across different alginate concentrations. The boxes represent the interquartile range (IQR), the lines indicate the median, whiskers extend to the data range, and individual data points displayed as circles are outliers. (**B**) Box-and-whisker plots showing percent swelling across different PVA concentrations, with statistical significance indicated between specific pairs of PVA concentrations. (**C**) Three-dimensional scatter plot illustrating the combined effect of alginate and PVA concentrations on percent swelling, providing an integrated view of their joint impact on swelling behavior. Asterisks denote statistical significance: * *p* < 0.05.

The 48-h swelling ratio of hydrogel samples was analyzed as a function of both alginate and PVA concentrations, revealing distinct trends in response to these variables (Figure 2). For varying alginate concentrations, the mean percent swelling ratios range from 147.6% (±60.1) at 5% alginate to 159.5% (±46.9) at 10% alginate. As the alginate concentration increases, the swelling values remain fairly stable, with a mean percent swelling value of 150.9% (±20.8) at 15% alginate, 154.6% (±21.0) at 20% alginate, and 149.7% (±21.5) at 25% alginate. Despite the range in swelling percentages, alginate concentration alone did not have a statistically significant effect on the swelling ratio (F = 0.614, *p* = 0.654), and pairwise comparisons confirmed the absence of significant differences between alginate concentrations. These results suggest that, within the tested range, alginate concentration has a relatively minor impact on the swelling behavior of the hydrogel matrix.

In contrast, PVA concentration showed a significant main effect on percent swelling across the different formulations independent of alginate concentration (F = 11.115, *p* < 0.001), highlighting PVA’s dominant role in influencing water uptake and retention. The mean percent swelling values range from 128.3% (±15.4) at 15% PVA to 172.5% (±61.2) at 0% PVA. This reduction in swelling with increasing PVA concentration was further confirmed by post hoc comparisons, which revealed that hydrogels containing 15% PVA exhibited significantly lower swelling than those with 0% and 5% PVA (both *p* < 0.001). This finding emphasizes that intermediate levels of PVA (around 15%) yield the most substantial reduction in swelling, highlighting the potential of these concentrations to modulate hydrogel hydration properties in controlled tissue-engineering applications (Figure 2B).

A significant interaction effect between alginate and PVA concentrations was observed (F = 5.650, *p* < 0.001), indicating that the influence of PVA concentration on swelling behavior is modulated by the specific level of alginate concentration present in the bioink formulation. The 3D plot in Figure 2C visually demonstrates this interaction, suggesting that higher PVA concentrations generally lead to lower swelling, while the alginate concentration exerts a subtler, secondary impact. This combined analysis underscores that, under the tested conditions, PVA concentration exerts a more pronounced effect on swelling behavior compared to alginate concentration.

**Figure 3:**
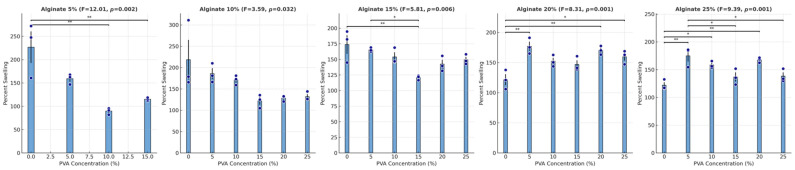
Percent swelling of hydrogel samples at varying concentrations of PVA with fixed alginate concentrations. Bar graphs represent mean percent swelling at different PVA concentrations for each specified alginate concentration. Error bars indicate the standard error of the mean (SEM). Individual data points are overlaid as navy circles, showing variability within each concentration group. Statistical significance between selected PVA concentration pairs is indicated by asterisks above the bars, with the following notation: * *p* < 0.05, ** *p* < 0.01, ANOVA F-statistic and *p*-value for each alginate concentration level are included in the title of each panel, highlighting significant differences in percent swelling across PVA concentrations within each fixed alginate concentration level.

Given the overall significance of PVA concentration on the 48-h swelling of the hydrogels, we further analyzed its effect within each fixed alginate concentration (Figure 3). When using 5% alginate, increasing the PVA concentration generally led to a reduction in swelling, with mean swelling decreasing from 226.7% at 0% PVA to 115.1% at 15% PVA, with the ANOVA results indicating a significant effect of PVA concentration on percent swelling (*p* = 0.002). This trend was observed across all but 10% alginate, with significant effects of PVA concentration on swelling found at 15% alginate (*p* = 0.006), 20% alginate (*p* = 0.001), and 25% alginate (*p* < 0.001).

At each alginate concentration, increases in PVA from 0% to 15% or to 20% typically resulted in lower swelling percentages, with the most pronounced reductions occurring between 10% and 15% PVA. Notably, across alginate levels, the reduction in swelling when PVA was added was most pronounced at intermediate PVA concentrations, specifically around 15% PVA.

**Figure 4:**
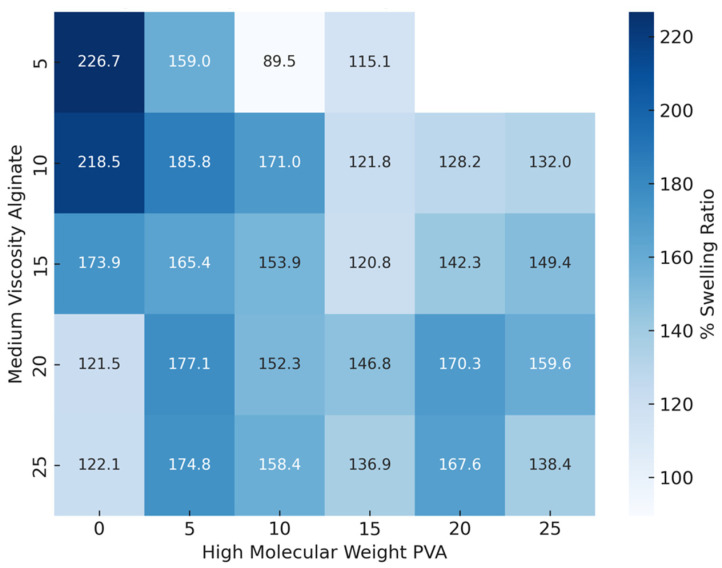
Heatmap displaying the 48-h percent swelling ratio of hydrogel samples across varying concentrations of high-molecular-weight PVA and medium-viscosity alginate. The color intensity corresponds to the swelling ratio, with darker blue shades representing higher swelling percentages. Each cell is annotated with the mean swelling ratio (%) for the corresponding combination of PVA and alginate concentrations. This visualization highlights the inverse relationship between PVA concentration and swelling, as higher PVA levels generally correspond to lower swelling ratios, particularly at lower alginate concentrations.

Figure 4 provides a comprehensive view of the interaction between the two bioink additives, illustrating the combined effects of PVA and alginate concentrations on the 48-h swelling ratio. As higher PVA levels are generally associated with lower swelling ratios, particularly at lower alginate concentrations, this visualization helps to summarize the collective impact of both components on hydrogel swelling behavior.

The results indicate a clear relationship between PVA concentration and the percent swelling across fixed alginate concentrations. For each alginate concentration, increasing the PVA concentration generally resulted in a reduction in percent swelling. The observed trend suggests that higher PVA concentrations result in a denser polymer network structure, which restricts water absorption and reduces swelling. Specifically, at the lower alginate concentrations, the effect of increasing PVA is more pronounced, as seen in the considerable drop in percent swelling when moving from lower to higher PVA concentrations. Conversely, at higher alginate concentrations, the swelling response becomes less sensitive to PVA changes, implying a potentially stronger influence of alginate on the network’s structural integrity, which may dominate over the effect of PVA. These findings highlight the synergistic role of PVA and alginate in controlling swelling behavior, where adjusting both concentrations could fine-tune the scaffold’s absorptive properties for specific applications.

In cartilage tissue engineering, the swelling behavior of hydrogels is a critical property as it influences both the mechanical integrity and the nutrient transport within the construct, which are essential for sustaining chondrocyte activity and promoting extracellular matrix formation. Ideal swelling properties for cartilage constructs generally involve moderate water uptake, which helps maintain the structural integrity while ensuring sufficient space for cell proliferation and ECM deposition. Interestingly, alginate concentrations did not significantly decrease hydrogel swelling as we had hypothesized. Previous research on alginate-based hydrogels has shown that increasing alginates leads to stable swelling ratios, providing a balance between flexibility and structural stability, favorable for cartilage tissue applications [21,22]; however, these studies used concentrations of alginate <5% and, thus, the stable swelling ratio seen in our study may be the result of higher starting concentrations.

Our findings did demonstrate that increasing PVA concentration significantly reduces swelling, indicating the formation of a denser polymer network that limits excessive hydration, potentially enhancing the stability of cartilage constructs. This aligns with previous studies that demonstrated that hydrogels with properly adjusted PVA levels show reduced swelling and improved load-bearing properties, essential for mimicking native cartilage’s compressive strength [23]. Hao et al. designed a PVA-based hydrogel for cartilage engineering using 10% PVA that exhibited resistance to swelling and high compressive strength [19]. Spiller et al. also demonstrated that semi-degradable PVA hydrogels maintained a balance between porosity and swelling, properties critical for integration with cartilage tissue, cell proliferation, and ECM production [24]. In comparison, our results highlight that intermediate PVA concentrations (e.g., 15%) show a balanced degree of swelling, suggesting a potential formulation for cartilage tissue engineering applications that require both hydration control and structural integrity.

### 2.3. Degradation

The degradation behavior of the hydrogel constructs after 28 days in culture was analyzed as a function of both alginate and PVA concentrations, revealing distinct patterns primarily influenced by PVA levels. For alginate concentration, the mean percent degradation values range from the lowest degradation of 6.6% (±7.7) at 20% alginate to the highest of 8.8% (±12.0) at 5% alginate. Alginate concentration did not exhibit a statistically significant main effect on degradation (F = 1.578, *p* = 0.185), indicating that variations in alginate levels alone do not significantly impact the degradation rate. Pairwise comparisons further confirmed the absence of significant differences between specific alginate concentrations, suggesting that within the tested range, alginate concentration alone has minimal impact on long-term degradation under the culture conditions used.

**Figure 5:**
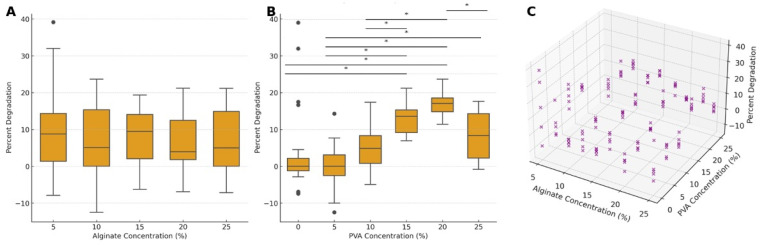
Percent degradation of constructs after 28 days in culture in response to varying concentrations of alginate and PVA. Box-and-whisker plots show percent degradation across alginate concentrations (**A**) and PVA concentrations (**B**), with boxes representing the interquartile range (IQR), and the lines indicating the median. Whiskers extend to the data range, and individual data points displayed as circles are outliers. A 3D scatter plot (**C**) illustrates the combined effects of alginate and PVA concentrations on percent degradation, offering an integrated view of their joint impact on material degradation. Asterisks denote statistical significance: * *p* < 0.05.

Conversely, PVA concentration showed a pronounced effect on degradation, demonstrating its critical role in modulating the stability of the hydrogel matrix. The mean percent degradation values ranged from the lowest degradation of −0.3% (±5.9) at 5% PVA to the highest of 16.8% (±3.0) at 20% PVA. A significant main effect of PVA concentration on degradation was observed (F = 19.111, *p* < 0.001), underscoring the substantial influence of PVA on degradation rates over time. Post hoc comparisons between PVA concentration groups revealed several statistically significant differences in percent degradation (Figure 5B). For example, 5% PVA led to significantly decreased degradation compared to 15%, 20%, and 25% PVA (*p* < 0.05); 15% PVA led to significantly higher degradation than 0%, 5%, and 10% PVA (*p* < 0.05); and 20% PVA exhibited a significant increase in percent degradation compared to 0%, 5%, 10%, and 25% PVA (*p* < 0.05). These findings illustrate that PVA concentration is a key determinant in the degradation behavior of hydrogels, with higher PVA levels correlating with increased degradation rates.

A significant interaction effect between alginate and PVA concentrations (F = 8.148, *p* < 0.001) highlights that degradation is influenced not only by the individual concentration effects of PVA but also by its combined interaction with alginate. Figure 5C illustrates the joint influence of alginate and PVA concentrations on percent degradation, offering a visual representation of how the degradation pattern varies based on the interplay of these two components. In general, higher PVA concentrations correlate with increased degradation, whereas alginate concentration exerts a comparatively moderate effect. This interaction suggests that while alginate contributes some degree of structural stability, PVA concentration plays a more prominent role in determining the material’s long-term breakdown, emphasizing the necessity to balance these components to achieve desired stability.

**Figure 6:**
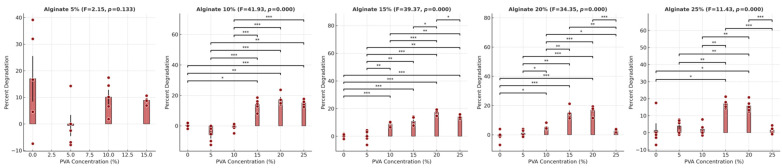
Percent degradation of hydrogel samples after 28 days, shown across varying PVA concentrations with fixed alginate concentrations. Each bar represents the mean degradation percentage for a given PVA concentration at a fixed alginate level. Error bars represent the standard error of the mean (SEM), and individual data points are displayed as circles, providing insights into data spread and individual variation. Statistical significance between selected PVA concentration pairs is indicated by asterisks above the bars, with * *p* < 0.05, ** *p* < 0.01, and *** *p* < 0.001. ANOVA F-statistic and *p*-value are included in each panel title, highlighting significant differences in degradation across PVA concentrations within each fixed alginate concentration level.

Given the significant effect of PVA concentration on the degradation of the hydrogels, we chose to further explore the relationship between PVA concentration and degradation within fixed alginate concentrations (Figure 6). At 5% alginate, there was no statistically significant effect of PVA on degradation; however, for alginate concentrations of 10% and higher, PVA significantly affected degradation. At 10%, 15%, 20%, and 25% alginate concentrations, increases in PVA concentration contributed to significant degradation increases (all *p* < 0.0001). These findings indicate that, except at the lowest alginate concentration (5%), increasing PVA concentration contributes to a statistically significant increase in degradation across all other alginate levels.

**Figure 7:**
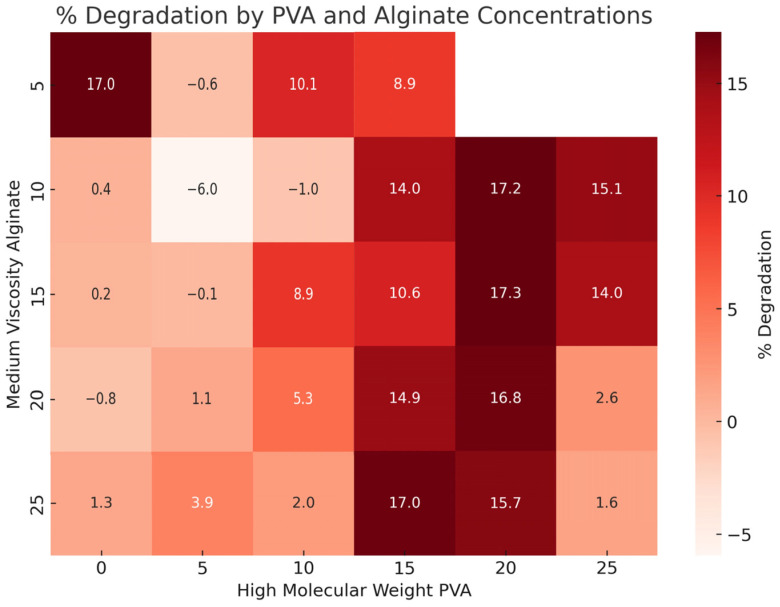
Heatmap displaying the percent degradation of hydrogel samples after 28 days across varying concentrations of high-molecular-weight PVA and medium-viscosity alginate. The color intensity corresponds to the degradation percentage, with darker red shades representing higher degradation levels. Each cell is annotated with the mean degradation percentage (%) for the respective combination of PVA and alginate concentrations.

The heatmap in Figure 7 provides a comprehensive view of the degradation behavior across varying PVA concentrations in alginate-based hydrogels. This trend highlights the inverse relationship between PVA concentration and matrix stability, where increased PVA may disrupt the polymer network, accelerating degradation over time. The data suggest that while higher PVA levels tend to increase degradation, the rate varies depending on the specific balance between PVA and alginate concentrations.

The impact of PVA concentration on degradation reveals a complex trend, particularly when combined with fixed alginate levels. Moderate PVA concentrations tend to stabilize the hydrogel, minimizing degradation, while higher PVA concentrations increase degradation, especially at the lower and intermediate alginate levels. For instance, at alginate concentrations of 10% and 15%, degradation rises significantly as PVA exceeds certain thresholds, suggesting that while moderate PVA levels reinforce structural stability, very high concentrations may disrupt the polymer network balance, leading to faster degradation. This insight highlights the importance of adjusting PVA concentration to achieve long-term stability, particularly in applications where controlled degradation is essential.

In practical applications, these results suggest that altering PVA concentration provides a more robust approach to modulating degradation, as certain levels of PVA alone or in combination with alginate can produce significantly different degradation outcomes. The observed synergy between alginate and PVA concentrations may offer valuable opportunities to tailor degradation rates in biomedical applications, particularly in scenarios where controlled material breakdown is essential for facilitating tissue regeneration.

In cartilage tissue engineering, achieving a balanced degradation rate is crucial. Controlled degradation provides space for cellular growth, ECM production, and nutrient diffusion while avoiding premature scaffold breakdown that could compromise structural integrity [25]. Our results show that increasing PVA concentration notably influences degradation, with 20% PVA leading to substantial degradation. While previous studies report mixed effects of PVA on hydrogel degradation, these inconsistencies may stem from variations in hydrogel composition and network interactions in non-alginate-based systems [4,23,24,26,27]. In alginate-based bioinks, intermediate PVA concentrations (e.g., 10–15%) appear to offer a balance between stability and degradation, which is necessary to support cartilage tissue formation.

This balance is critical; too rapid degradation compromises structural function, hindering regeneration, while too slow degradation impedes cell proliferation and nutrient diffusion. Fine-tuning PVA concentration is, therefore, essential to influence the mechanical and physical properties of hydrogels designed for cartilage repair. The effect of PVA concentration on degradation highlights its use in balancing material stability. At a low PVA concentration of 5%, degradation is minimized, suggesting stability over time; however, the swelling of the construct remains high at this level, indicating that while degradation is controlled, water absorption is still substantial. A higher PVA concentration of 15% significantly reduces swelling but increases degradation, potentially compromising long-term stability.

A balanced option of 10% PVA offers moderately reduced swelling and manageable degradation, providing a stable polymer matrix suitable for applications requiring both minimized degradation and reduced swelling. Thus, while 15% PVA is ideal for swelling control and 5% PVA for reducing degradation, 10% may serve as an ideal middle ground for tissue engineering. Overall, these findings show the ability to tailor material concentrations to have desired properties for diverse biomedical applications.

### 2.4. Mechanical Testing

**Figure 8:**
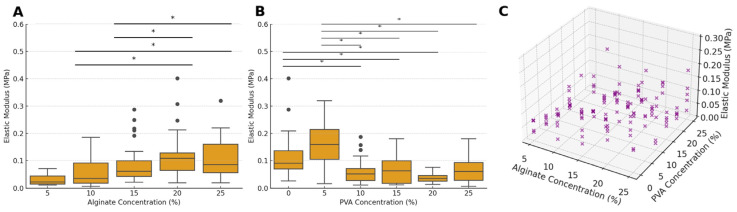
Elastic modulus behavior of hydrogel samples in response to varying alginate and PVA concentrations. (**A**) Box-and-whisker plots of modulus values across alginate concentrations, with boxes representing the interquartile range (IQR), the lines indicating the median, and whiskers extending to the data range, with outliers represented as individual data points. (**B**) Box-and-whisker plots of modulus values across different PVA concentrations, with significant differences between specific PVA concentrations. (**C**) Three-dimensional scatter plot illustrating the combined effect of alginate and PVA concentrations on modulus, offering an integrated view of their joint impact on mechanical properties. Asterisks denote statistical significance: * *p* < 0.05.

The analysis of the elastic modulus at various alginate and PVA concentrations shows inverse trends in their effect on mechanical strength of the hydrogels (Figure 8). For alginate concentrations, the mean elastic modulus values range from 0.03 MPa (±0.02) at 5% alginate to 0.12 MPa (±0.09) at 20% alginate. As the alginate concentration increases, the modulus generally rises, with mean values of 0.06 MPa (±0.05) at 10% alginate, 0.09 MPa (±0.07) at 15% alginate, and 0.11 MPa (±0.07) at 25% alginate. There is a statistically significant main effect of alginate concentration on the elastic modulus (F = 15.32, *p* < 0.001) independent of PVA concentrations, with post hoc comparisons revealing significant differences between lower alginate concentrations (5% and 10%) and higher concentrations (20% and 25%) (*p* < 0.05). These results suggest that increasing alginate concentration enhances the stiffness of the hydrogel matrix, likely due to the increased crosslinking density and polymer network stability provided by higher alginate levels.

In contrast, PVA concentration exhibited an inverse relationship with the elastic modulus, particularly at higher concentrations. Mean modulus values range from 0.12 MPa (±0.09) at 0% PVA to 0.06 MPa (±0.05) at both 10% and 15% PVA, indicating a decrease in modulus as PVA is introduced, with another drop to 0.04 MPa (±0.02) at 20% PVA. There was a significant main effect of PVA concentration on modulus (F = 14.37, *p* < 0.001), showing that PVA levels considerably influence hydrogel strength independent of alginate concentrations. Post hoc comparisons showed significant differences in the elastic modulus between several PVA concentration pairs (e.g., 0% vs. 10%, 5% vs. 20%, *p* < 0.05) (Figure 8B), indicating that the addition of PVA, particularly at moderate levels, generally results in a lower modulus compared to pure alginate.

A significant interaction effect was observed between alginate and PVA concentrations (F = 3.36, *p* < 0.001), suggesting that the effect of PVA concentration on the elastic modulus is influenced by alginate concentration levels. The 3D scatter plot in Figure 8C illustrates this interaction, showing that while both alginate and PVA concentrations contribute to the elastic modulus, PVA concentration plays a more pronounced role at lower alginate levels.

**Figure 9:**
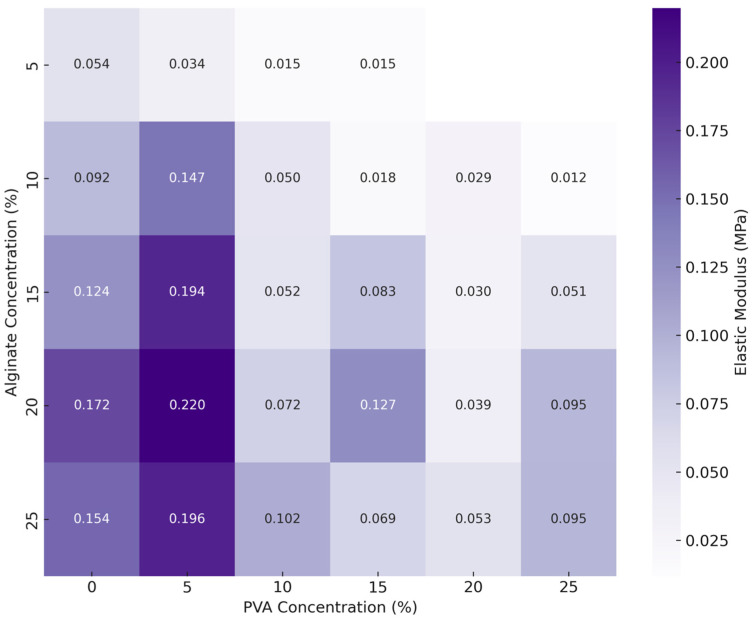
Heatmap of elastic modulus by PVA and alginate concentrations. This heatmap illustrates the elastic modulus (MPa) of constructs at varying concentrations of PVA and alginate, as determined by compression testing. Each cell represents the mean elastic modulus for a specific combination of PVA and alginate concentrations, with values shown to three decimal places. Darker shades of purple indicate higher elastic modulus values, signifying stronger constructs.

Interestingly, when examining individual formulations of composite hydrogels, those with PVA concentrations in the range of 10% to 25% were overall less stiff than pure alginate hydrogels. The addition of 5% PVA, however, provided the most benefit in terms of enhancing the mechanical strength across most alginate concentrations (Figure 9). This finding indicates that lower concentrations of PVA can reinforce the alginate matrix without significantly compromising stiffness, suggesting that a low PVA level may be ideal when both strength and flexibility are required.

These results indicate a complex relationship between both alginate and PVA concentrations and the elastic modulus of the hydrogels, suggesting that each component contributes differently to the mechanical strength. Specifically, increasing alginate concentration significantly enhances the modulus, likely due to the increased crosslinking density and stability in the polymer matrix associated with higher alginate levels. This aligns with the findings of Shams et al., who reported that higher sodium alginate concentrations led to an increased storage modulus, resulting in greater hydrogel strength, which is beneficial for load-bearing applications in tissue engineering [28].

In contrast, increasing PVA concentration generally leads to a softer hydrogel network, reducing the elastic modulus compared to pure alginate formulations. While PVA has been investigated for its potential to enhance mechanical properties in certain applications by tuning the crosslinking density [7,29], our results suggest that adding PVA at concentrations above 5% does not enhance stiffness of alginate gels. Instead, composite hydrogels with PVA concentrations between 10% and 20% were generally less stiff than pure alginate hydrogels at higher concentrations (20% and 25%).

Yumin et al. observed a similar nonlinear trend in that the addition of up to 2% PVA to an alginate hydrogel increased tensile strength but concentrations above 4% resulted in decreased tensile strength [30]. Similarly, a computational study on PVA–alginate blends reported that alginate hydrogels without PVA exhibited a higher tensile modulus, with the addition of PVA leading to a stepwise decrease in tensile modulus [18]; however, the study also reported an increased Cauchy pressure, or ductility of the hydrogel as PVA concentrations increased. It is important to note that while a direct comparison of mechanical properties of hydrogels in this study to those in the literature is unfounded due to variations in hydrogel preparation methods, components, and mechanical testing parameters, these findings still underscore the nuanced mechanical behavior of alginate–PVA blends.

Interestingly, the combination of 5% PVA with 20% alginate yielded the highest elastic modulus among the tested formulations, indicating that this particular blend offers a balance of alginate’s stiffness with the addition of a minimal yet supportive concentration of PVA. This specific combination may achieve a structural configuration that reinforces mechanical strength without overly compromising flexibility, making it potentially suitable for applications where both rigidity and some degree of elasticity are required.

In cartilage tissue engineering, mechanical strength is critical as the hydrogel scaffold must support load-bearing functions while maintaining structural integrity to be viable for translational applications. Articular cartilage exhibits complex mechanical properties under dynamic loading conditions with the equilibrium and Young’s moduli around 0.5 MPa [31,32,33]. While cartilage’s behavior under real-life conditions is more complex, there is still the need for engineered hydrogels that can mimic these viscoelastic and mechanical properties [34]. Our hydrogel formulated with 5% PVA and 20% alginate hydrogel, with an elastic modulus of 0.22 MPa, approaches this target range, suggesting suitability for load-bearing applications. The rigidity from the higher alginate and flexibility from the low PVA may help mimic the strength–resilience balance in native cartilage, essential for withstanding repetitive loading. Prior research has also shown that encapsulating chondrocytes in bioinks with 20% alginate, 5% PVA, and ECM powder enhances viability and gene expression [8], and our findings further support the potential of this formulation for cartilage tissue engineering.

Overall, these results show that varying alginate-to-PVA ratios contributes distinct effects on the 3D-printed hydrogel. Higher alginate concentrations increase the ionic crosslinking ability of the gel, resulting in a denser and more rigid network that enhances structural integrity. Alternatively, increasing PVA concentrations introduces hydrogen bonding and crystalline regions, promoting elasticity and reducing swelling by restricting water absorption. However, extreme ratios of components could destabilize the gel as seen in this study. Higher PVA concentrations tend to result in diminished stiffness and accelerated degradation, likely due to disrupted polymer interactions of the alginate.

Selecting the proper ratio of alginate and PVA for extrusion bioprinting is critical to achieve a hydrogel with balanced properties suitable for its intended application. While alginate primarily drives stiffness, PVA fine-tunes elasticity and toughness. Higher PVA concentrations are suited for flexibility, whereas higher alginate levels provide structural rigidity. This tunable interplay allows for customization of the swelling, degradation, and mechanical properties, offering a promising approach to designing hydrogels conducive to cellular growth and extracellular matrix deposition, as required in cartilage tissue engineering.

Although this study was conducted in the context of cartilage tissue engineering, driven by the desire to develop advanced bioinks for cartilage regeneration, the materials and methodologies presented here can be adapted for a wide range of tissue-engineering applications. The biocompatibility and tunable properties of the alginate–PVA bioinks make them suitable for applications such as bone regeneration or other load-bearing tissues, highlighting the versatility of these materials.

## 3. Conclusions

The comprehensive analysis of the physical and mechanical properties of alginate and PVA bioinks performed in this study provides a framework for selecting the proper bioink formulation for applied 3D tissue engineering. Our findings indicate that PVA concentration is a primary factor in controlling alginate hydrogel swelling, with intermediate concentrations (10–15%) effectively reducing water absorption. Degradation analysis indicated that higher PVA levels accelerated alginate hydrogel breakdown, while 5% PVA maintained lower degradation rates, suggesting that PVA concentration can be adjusted to achieve the desired stability. Alginate concentration predominantly affected the elastic modulus, with higher alginate levels (e.g., 20%) significantly enhancing stiffness. The formulation of 5% PVA and 20% alginate yielded an elastic modulus of 0.22 MPa, approaching values necessary to approximate cartilage’s mechanical properties. Overall, these results highlight the importance of tuning alginate and PVA concentrations to balance swelling, degradation, and mechanical strength, providing a framework for developing bioinks tailored to cartilage tissue engineering.

## 4. Materials and Methods

### 4.1. Synthesis of Experimental Bioink Formulations

Bioinks were created with varying compositions of additives including five different concentrations (5% *w*/*v*, 10% *w*/*v*, 15% *w*/*v*, 20% *w*/*v*, and 25% *w*/*v*) of medium-viscosity alginate (A2033, Sigma-Aldrich, St. Louis, MO, USA); six different concentrations (0% *w*/*v*, 5% *w*/*v*, 10% *w*/*v*, 15% *w*/*v*, 20% *w*/*v*, and 25% *w*/*v*) of Polyvinyl alcohol (PVA) powder (P8136, Sigma-Aldrich, St. Louis, MO, USA); and 5% *w*/*v* gum arabic (51198, Sigma-Aldrich, St. Louis, MO, USA) as an emulsifier. To mix, PVA was first added into aqueous solution followed by gum arabic and alginate powers and stirred until homogenous solution was obtained. The bioinks were subsequently loaded into a 3 cc syringe for immediate printing.

### 4.2. Bioprinting of Alginate–PVA Constructs

An in-house bioprinter (BIO X™, Cellink, Gothenburg, Sweden) was utilized to print the alginate–PVA hydrogel scaffolds of 10 mm H × 10 mm D in dimension via the use of a 3 cc syringe with varying gauge nozzles (16–22 G) based on bioink viscosity (Table 1). Of note, bioinks consisting of 5% alginate with both 20% and 25% PVA were too viscous to mechanically extrude with a 16 G nozzle and, thus, were not characterized in this study.

**Figure 10:**
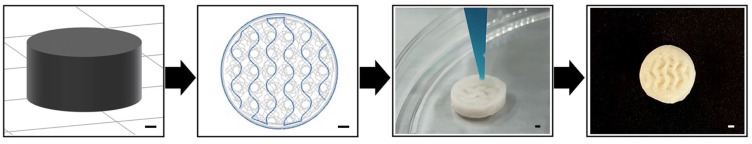
Schematic representation of the bioprinting process for alginate–PVA hydrogel constructs. From left to right: (1) Three-dimensional model of the cylindrical construct designed for bioprinting; (2) slicing of the 3D model to create a 50% gyroid infill pattern; (3) extrusion of the hydrogel bioink in the specified design; (4) final printed hydrogel construct after crosslinking. Scale bar represents 1 mm.

The hydrogel structures were produced with a grid lattice infill using the previously outlined bioink compositions above (Figure 10). After printing, the constructs were crosslinked by submersion in 0.5 M calcium chloride (CaCl_2_) for 30 min. Fifteen samples were printed within each group. For each group, the constructs underwent either culture for swelling (n = 5) and degradation (n = 5) analysis or immediate mechanical testing (n = 5).

### 4.3. Swelling Analysis

To determine the swelling behavior of constructs in culture conditions, we evaluated the ratio of swelling of the hydrogels after 48 h in culture compared to their initial swelling. Five constructs from each group were fabricated and immediately weighed to provide the initial as-printed wet weight (*W_i_*). The samples were stored overnight at −80 °C to freeze, then lyophilized for 24 h at −50 °C and 0.2 mBar. Subsequently, the samples were weighed to obtain the dry weight (*W_di_*). The samples were then cultured for 48 h in standard culture medium. The swollen gels were removed from culture after 48 h, blotted to remove excess water, and weighed to provide the 48-h wet weight (*W*_48_). These samples were then stored overnight at −80 °C to freeze, lyophilized for 24 h at −50 °C and 0.2 mBar, and weighed to obtain the 48-h dry weight (*W_d_*_48_). The percent swelling ratio (%) of the hydrogels at 48 h was calculated according to Equation (1) [35].

(1) $$48\;h\;Swelling\;Ratio\;(\%)=\frac{\left(\frac{W_{48}\;-\;W_{d48}}{W_{d48}}\right)}{\left(\frac{W_{i}\;-\;W_{di}}{W_{di}}\right)}\times 100$$

### 4.4. Degradation of Hydrogel Constructs in Standard Culture Medium

Degradation characteristics of the hydrogels with varying concentrations of alginate–PVA in standard culture conditions were obtained by printing, then immediately weighing the samples to obtain the initial as-printed wet weight. After recording the initial wet weight, the constructs were placed in standard culture medium (high glucose Dulbecco’s Modified Eagle Medium [DMEM] containing 10% fetal bovine serum, 100 U/mL penicillin, 100 µg/mL streptomycin, 2.5 µg/mL Fungizone) and cultured at 37 °C and 5% CO_2_. After days 1, 14, and 28 (n = 5, each time point), the hydrogels were transferred into 15 mL Falcon tubes, then weighed to obtain the remaining wet weight. The remaining hydrogel mass was determined by normalizing the final wet weight from each time point in standard culture conditions to the as-printed initial wet weight of each respective construct.

### 4.5. Mechanical Testing

**Figure 11:**
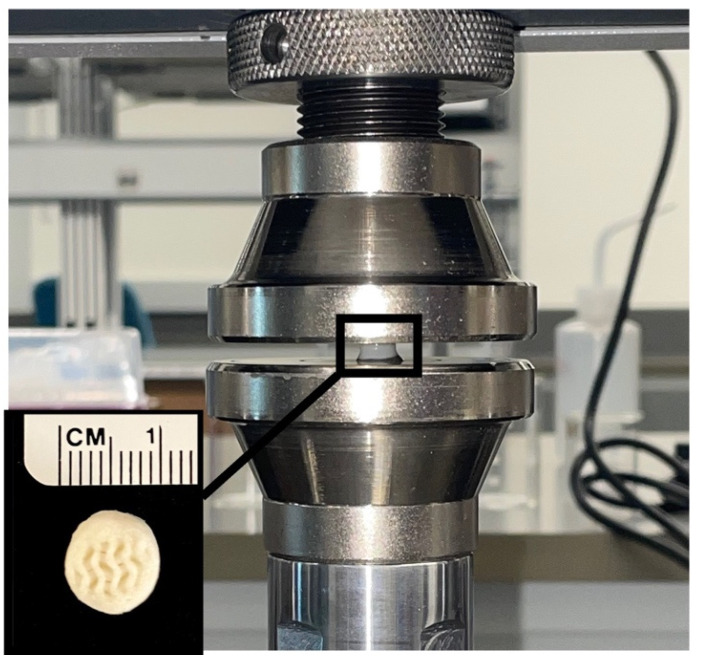
Uniaxial unconfined compression testing setup. A cylindrical 3D bioprinted hydrogel sample is positioned between stainless-steel compression platens in the mechanical testing machine. The close-up inset shows the hydrogel sample’s gyroid infill structure with a scale for size reference. True measurements of the hydrogels were performed using digital calipers prior to compression testing.

The three-dimensional bioprinted samples were post-processed using a custom-designed slicing tool to ensure flat, parallel surfaces. The compressive properties of the hydrated scaffolds were evaluated through uniaxial unconfined compression tests using a Test Resources 300 series universal testing machine equipped with a 100 N load cell. Five replicates (n = 5) of cylindrical 3D-printed hydrogels were tested within each group. The height and diameter of the sample were measured using digital calipers prior to loading the sample on the stainless-steel platen (Figure 11). Compression loading was performed at a rate of 0.5 mm min^−1^ and continued until 15% strain was achieved. The Young’s modulus of the scaffolds was determined as the slope in the linear-elastic deformation region of stress–strain diagrams, between 5% and 10% strain, using custom-designed MATLAB scripts. For volume calculations, the printed constructs were assumed to be perfectly cylindrical; thus, the cross-sectional area of the sample was modeled as a fixed circle. A semi-automated process was utilized to isolate the elastic region of the stress–strain curves and generate a linear relationship. The coefficient of the first-degree polynomial corresponded to the value of the elastic modulus and is reported in MPa.

### 4.6. Statistical Analysis

Statistical analyses were performed using Microsoft Excel (version 16.90.2) and GraphPad Prism (version 10.3.01). Descriptive statistics, including means and standard deviations, were calculated for each experimental condition. To examine the effects of alginate and PVA concentrations on swelling, degradation, and the elastic modulus, two-way Analysis of Variance (ANOVA) tests were conducted, treating alginate and PVA as independent variables. Where significant main effects or interactions were detected, Tukey’s post hoc test was applied to identify pairwise differences between concentration levels. Additionally, to examine the effects of varying PVA concentrations within set concentrations of alginate, a one-way ANOVA was performed followed by Tukey’s post hoc test to identify significant pairwise differences across PVA concentrations within a specific alginate concentration. Normality of data was assessed with the Shapiro–Wilk test, and elastic modulus data were analyzed within linear-elastic deformation regions (5–10% strain) using a custom MATLAB (R2024b, MathWorks, 2024) script. All *p*-values less than 0.05 were considered statistically significant (*p* < 0.05).

## Figures and Tables

**Table 1:** Nozzle sizes selected for 3D bioprinting based on varying concentrations of medium-viscosity alginate and polyvinyl alcohol (PVA) in bioink formulations. Each cell represents the nozzle gauge chosen to achieve peak printability for the corresponding combination of PVA and alginate concentrations, as determined by the resulting bioink viscosity.

		High-Molecular-Weight PVA					
		0%	5%	10%	15%	20%	25%
Medium-Viscosity Alginate	5%	22 G	20 G	20 G	18 G	16 G	
	10%	22 G	22 G	20 G	18 G	18 G	16 G
	15%	22 G	22 G	20 G	18 G	18 G	18 G
	20%	22 G	22 G	20 G	18 G	18 G	18 G
	25%	22 G	22 G	20 G	18 G	18 G	18 G

## Data Availability

The raw data supporting the conclusions of this article will be made available by the authors on request.
